# Immunogenicity and safety of two-dose SARS-CoV-2 vaccination *via* different platforms in kidney transplantation recipients

**DOI:** 10.3389/fimmu.2022.951576

**Published:** 2022-09-16

**Authors:** Chien-Chia Chen, Yi-Jen Huang, Mei-Jun Lai, Min-Huey Lin, Wei-Chou Lin, Hui-Ying Lin, Yu-Chun Lin, Yu-Tsung Huang, Ya-Fen Lee, Meng-Kun Tsai, Chih-Yuan Lee

**Affiliations:** ^1^ Department of Surgery, National Taiwan University Hospital, Taipei, Taiwan; ^2^ Department of Pharmacy, National Taiwan University Hospital, Taipei, Taiwan; ^3^ Department of Laboratory Medicine, National Taiwan University Hospital, Taipei, Taiwan; ^4^ Department of Nursing, National Taiwan University Hospital, Taipei, Taiwan; ^5^ Department of Pathology, National Taiwan University Hospital, Taipei, Taiwan; ^6^ Division of General Surgery, Department of Surgery, National Taiwan University Biomedical Park Hospital, National Taiwan University Hospital Hsinchu Branch, Hsinchu, Taiwan

**Keywords:** SARS-CoV-2, vaccines, platform, immunogenicity, kidney transplant

## Abstract

After kidney transplantation, patients exhibit a poor response to severe acute respiratory syndrome coronavirus 2 (SARS-CoV-2) vaccination. However, the efficacy and adverse effects of vaccines based on different platforms in these patients remain unclear. We prospectively analyzed both anti-spike protein antibody and cellular responses 1 month after the first and second doses of SARS-CoV-2 vaccines in 171 kidney transplant patients. Four vaccines, including one viral vector (ChAdOx1 nCov-19, n = 30), two mRNA (mRNA1273, n = 81 and BNT162b2, n = 38), and one protein subunit (MVC-COV1901, n = 22) vaccines were administered. Among the four vaccines, mRNA1273 elicited the strongest humoral response and induced the highest interferon-γ levels in patients with a positive cellular response against the spike protein. Antiproliferative agents were negatively associated with both the antibody and cellular responses. A transient elevation in creatinine levels was noted in approximately half of the patients after the first dose of mRNA1273 or ChadOx1, and only one of them presented with borderline cellular rejection without definite causality to vaccination. In conclusion, mRNA1273 had better immunogenicity than the other vaccines. Further, renal function needs to be carefully monitored after vaccination, and vaccination strategies should be tailored according to the transplant status and vaccine characteristics.

## Introduction

Since the coronavirus disease 2019 (COVID-19) pandemic began in December 2019, several vaccines against its etiological agent, severe acute respiratory syndrome coronavirus 2 (SARS-Co-V-2), have been developed using different platforms (1, 2). A coordinated immune response is essential to control SARS-Co-V-2 (2, 3), and T cells are important in the fight against virus variants (4). However, several reports have shown impaired humoral and cellular immunity responses after vaccination in kidney transplant recipients (KTRs) (5, 6). All KTRs need to be fully vaccinated, and a third (7, 8) or fourth (9, 10) booster dose should be considered because of their immunocompromised status.

Owing to the availability of vaccines, most studies have focused on the results of messenger RNA (mRNA)-based vaccines, including BNT162b2 and mRNA1273, which are superior to vector vaccines (11) in the general population. For KTRs, evidence of immunogenicity comparing the different platforms is limited. A comparison between the mRNA and vector vaccines in 40 solid organ transplant recipients (12) revealed that the mRNA vaccines induced a greater antibody response than the vector vaccines, which resulted in more cellular activity than the former. A further comparison of BNT162b2 and mRNA1273 in KTRs showed that mRNA1273 had a higher seroconversion rate than BNT162b2 (13, 14). Notably, there have been cases of acute rejection in a KTR (15) and pancreas allograft rejection after widespread mRNA and vector vaccination (16). Both types of vaccines have good efficacy in promoting potent immune responses (17), which might be a concern in KTRs, especially when repeated doses are mandatory in this special group.

In addition to mRNA and vector vaccines, there are other platforms, including inactivated virus and protein subunits. The inactivated vaccine has a low antibody response rate (18), and the IgG response is approximately one-tenth that of the mRNA vaccine (19). Nevertheless, there is limited knowledge regarding protein subunit vaccines administered to KTRs. In Taiwan, a protein subunit vaccine, MVC-COV1901 (20), has been administered concurrently with mRNA1273, BNT162b2, and ChadOx1. The selection of vaccines depends on priorities according to the national policy and personal choice. In this prospective observational study, we analyzed both antibody and cellular responses after the first and second doses in KTRs to compare the four different vaccines. In addition to immunogenicity, we compared renal function changes after vaccination, which is a special concern with KTRs. These results could be applied to future vaccination strategies and development.

## Materials and methods

This prospective observational study was approved by the Research Ethics Committee of the National Taiwan University Hospital (NTUH: 202106046RINA).

### Patients

From June 2021, general administration of the SARS-Co-V-2 vaccine in Taiwan began with a homogenous two-dose regimen. Initially, ChAdOx1 and mRNA1273 were the only two available vaccines. In late August and early September, MVC-COV1901 and BNT162bs, respectively, were added to the vaccine list. KTRs without a COVID-19 history at NTUH were recruited for this observational study. After obtaining informed consent, blood samples were collected before (if available) and 28–42 days after each of the first and second doses. Spike protein-specific T cell stimulation was performed on the same day as collection. Plasma was isolated and frozen in batches for the anti-spike protein antibody test. Owing to the low prevalence of COVID-19 in Taiwan, anti-nucleocapsid antibodies were analyzed once using the first blood sample of each patient to exclude previous infections. Clinical data, including patient demographic profiles, graft function, and regular laboratory results, were reviewed.

### Quantification of T cell response after vaccination

The spike protein-specific T cell response was determined using a SARS-CoV-2 interferon (IFN)-γ release assay (IGRA) kit (Quan-T-Cell SARS-CoV-2, Euroimmun Medizinische Labordiagnostika, Luebeck, Germany). Whole blood collected in lithium heparin tubes was stimulated in blank, spike protein-, and mitogen-coated tubes for 16 h. The samples were then centrifuged to isolate and freeze the stimulated plasma, which was then subjected to an IFN-γ assay by ELISA according to the manufacturer’s protocol. The T cell response capacity was determined by subtracting the IFN-γ concentration (mIU/mL) in the test tube from that in the blank tube. According to the manufacturer, a value of >100 was regarded as a detectable response.

### Quantification of antibodies

The antibody concentration was determined using an electrochemiluminescence immunoassay kit for the spike protein and nucleocapsid protein (Elecsys Anti-SARS-CoV-2 S and Elecsys Anti-SARS-CoV-2, Roche) using a Cobas e 411 analyzer. Plasma was incubated with biotinylated and ruthenylated target antigens for 9 min. Streptavidin-coated microparticles were added for another 9 min incubation. Measurements were conducted using a photomultiplier. An antibody titer ≥0.8 U/mL was considered reactive according to the manufacturer’s instructions, and the upper limit titer was set to 5,000 U/mL in our laboratory. The Elecsys unit (U/mL) can be transformed into a binding antibody unit (BAU/mL), determined by the WHO, using the equation U = 0.972 × BAU.

### Data analysis

All numbers are presented as the mean ± standard deviation. Data among the four vaccine groups were compared using the chi-square test for categorical variables and ordinary one-way ANOVA or Kruskal-Wallis test for continuous variables. The correlation between the immune response and patient factors was analyzed based on the non-parametric Spearman correlation. For propensity score matching, we matched the patients based on their age in a 1:1 ratio to reduce selection bias with a standardized mean difference of 0.1. Using a two-tailed test, P<0.05 was considered a significant difference between groups. Statistical analysis was performed using GraphPad Prism 9.3.1 (GraphPad Software, LLC, CA, USA) and SPSS 28.0.1.1 (IBM Corp., Armonk, NY, USA)

## Results

### Patient demographic data

From July to December 2021, 167 KTRs vaccinated with at least one dose of the SARS-Co-V-2 vaccine were recruited for this study (**Table 1**). According to the vaccines, there were 30, 81, 38, and 22 patients vaccinated with ChAdOx1, mRNA1273, BNT162b2, and MVC-COV1901, respectively. Owing to vaccination policy and personal preference, KTRs vaccinated with mRNA1273 were older and had longer post-transplant intervals. There was no difference in the pre-vaccination renal function and immunosuppression regimens among the four groups. Tacrolimus with mycophenolate mofetil (MMF) and prednisolone were administered to most patients. The average tacrolimus level was approximately 4–6 ng/mL, and the daily MMF dose was approximately 1 g/day.

**Table 1:** Baseline characteristics among patients administered the four vaccines.

	ChAdOx1 (n=30)	mRNA1273 (n=81)	BNT162b2 (n=38)	MVC-COV1901 (n=22)	*P*-value
Age (years)	49.09 ± 11.94	60.29 ± 8.70	48.87 ± 11.98	46.97 ± 11.64	<0.0001
Male, n (%)	15 (50.00)	32 (39.51)	17 (44.74)	7 (31.82)	0.5644
Time since transplant (years)	6.23 ± 5.19	10.84 ± 8.21	8.32 ± 6.94	8.72 ± 7.09	0.0252
Creatinine (mg/dL)	1.23 ± 0.38	1.24 ± 0.58	1.17 ± 0.31	1.44 ± 0.45	0.8346
Maintenance IS, n (%)					0.8071
Calcineurin inhibitor	30 (100)	71 (87.65)	38 (100)	20 (90.91)	
Tacrolimus	28 (93.33)	60 (74.07)	37 (97.37)	18 (86.36)	
Level (ng/mL)	5.90 ± 2.07	4.71 ± 1.65	5.09 ± 1.37	4.64 ± 1.32	
Cyclosporine	2 (6.67)	11 (13.58)	1 (2.70)	2 (9.09)	
Level (ng/mL)	47.90 ± 46.53	61.35 ± 21.72	84.10	86.30 ± 100.83	
mTOR inhibitor	14 (46.67)	48 (59.26)	13 (34.21)	10 (45.45)	
Sirolimus	14 (46.67)	47 (58.02)	13 (34.21)	10 (45.45)	
Level (ng/mL)	1.45 ± 0.93	1.80 ± 1.75	1.18 ± 0.47	2.42 ± 2.68	
Everolimus	0	1 (1.23)	0 (0)	0 (0)	–
Level (ng/mL)	–	3.1	–	–	–
MMF	26 (86.67)	59 (72.83)	34 (89.47)	19 (86.36)	
Dose (g/day)	1.03 ± 0.35	1.01 ± 0.36	0.92 ± 0.35	1.05 ± 0.43	
Prednisolone	27 (90)	61 (75.31)	32 (84.21)	17 (77.27)	
Dose (mg/day)	3.92 ± 2.01	4.34 ± 2.17	3.95 ± 1.52	3.52 ± 2.40	

IS, immunosuppressant; MMF, mycophenolate mofetil.

### Immune responses after SARS-CoV-2 vaccination

**Figure 1:**
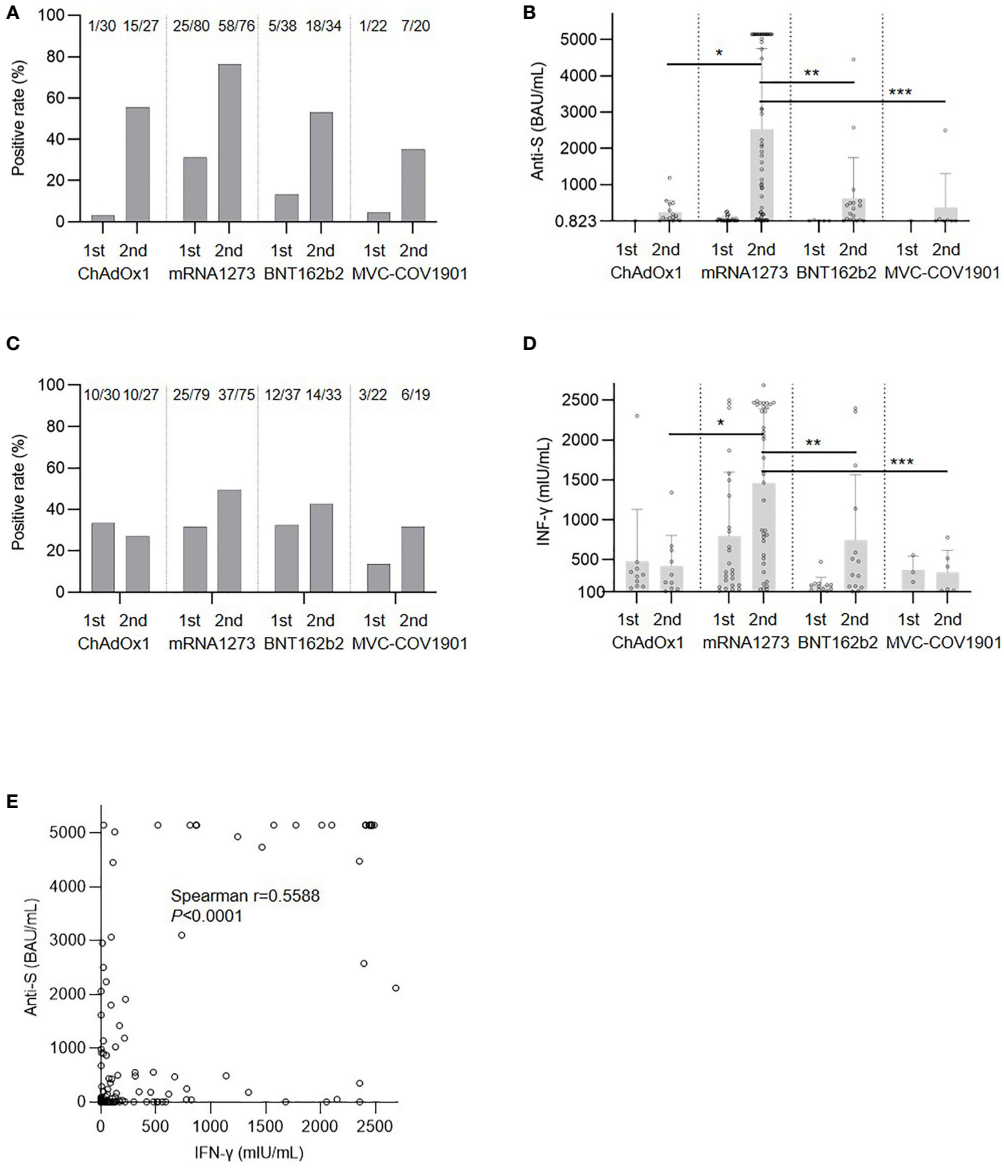
Immune response after severe acute respiratory syndrome coronavirus 2 (SARS-CoV-2) vaccination. **(A)** Response rate of antibody. **(B)** Anti-spike protein antibody (anti-S) level for positive patients; *P=0.0099, **P=0.0178, ***P=0.0025. **(C)** Cellular response rate. **(D)** Interferon-γ (IFN-γ) level for positive patients, *P=0.0115, **P=0.0414, ***P=0.0180. **(E)** Correlation between anti-S and IFN-γ levels.

The humoral response rates after the first and second doses of the SARS-CoV-2 vaccine are presented in **Figure 1A**. At least 28 days after the first dose, patients in the mRNA1273 group had the highest proportion of positive anti-spike protein antibodies (3%, 31%, 13%, and 5% for ChAdOx1, mRNA1273, BNT162b2, and MVC-COV1901, respectively; P=0.0009). For the second dose, the mRNA1273 group had the highest seropositive rate (56%, 76%, 53%, and 35%; P=0.0019) and mean antibody titer (**Figure 1B**) for the positive patients (244.89 ± 318.58, 2525.71 ± 2229.47, 616.91 ± 1128.31, and 368.60 ± 939.10 BAU/mL; P<0.0001). The cellular response rates were 33%, 32%, 32%, and 14% for ChAdOx1, mRNA1273, BNT162b2, and MVC-COV1901, respectively (P=0.3647; **Figure 1C**) after the first dose and 37%, 49%, 42%, and 32% after the second dose (P=0.4555). For patients with a positive response, the mRNA1273 group had the highest mean IFN-γ concentration (422.29 ± 382.15, 1460.30 ± 931.19, 746.54 ± 819.49, and 344.50 ± 274.51 mIU/mL for ChAdOx1, mRNA1273, BNT162b2, and MVC-COV1901, respectively; P=0.0004; **Figure 1D**).

Patients in the mRNA1273 group were older than those in the other groups and this might have had an effect on the immune response. We then used propensity score matching by age to compare mRNA1273 to ChAdOx1 (**Supplemental Table 1**), BNT162b2 (**Supplemental Table 2**), and MVC-COV1901 (**Supplemental Table 3**). After matching, mRNA1273 still showed a superior effect on both humoral and cellular immunity compared to the other vaccines. Similar to the results of a previous study (21), there was a positive correlation between humoral and cellular responses after the second dose (**Figure 1E**).

### Factors associated with the immune response

**Figure 2:**
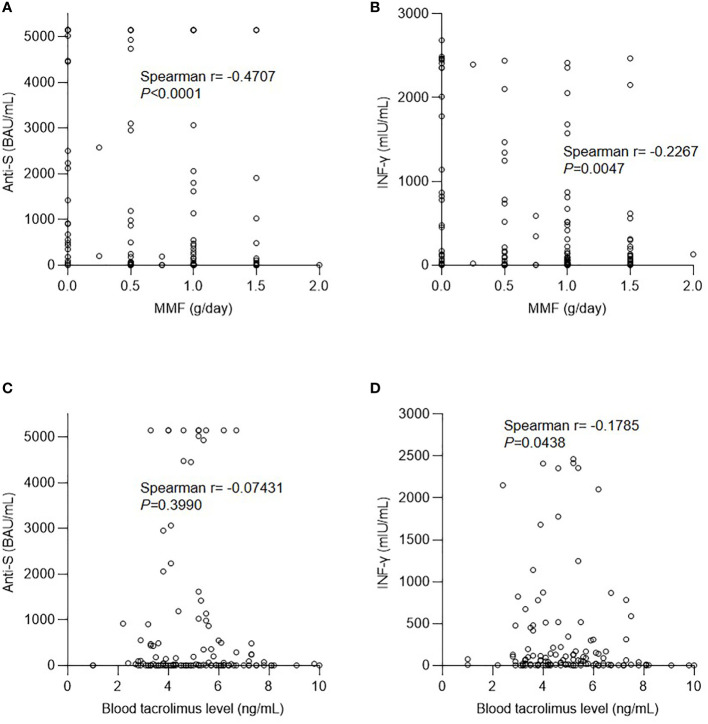
Effect of immunosuppressants on the immune response after vaccination. **(A)** Correlation between anti-spike protein antibody (anti-S) level and mycophenolate mofetil (MMF) dose. **(B)** Correlation between interferon-γ (IFN-γ) level and MMF dose. **(C)** Correlation between anti-S level and tacrolimus dose. **(D)** Correlation between IFN-γ level and tacrolimus dose.

Anti-metabolites are a negative factor for both antibody and cellular responses after SARS-CoV-2 vaccination in solid organ transplant recipients (22–24) and immunosuppressed patients being treated for immune-mediated diseases (25, 26). In our cohort, the daily dose of MMF was negatively correlated with the anti-S antibody level after the second dose (**Figure 2A**). The cellular response to the IFN-γ concentration was also negatively correlated with the MMF dose (**Figure 2B**). In contrast, tacrolimus levels were not correlated with humoral, but were mildly correlated with cellular, responses (**Figures 2C, D**). In addition to immunosuppressants, low CD3 and CD4 T cell counts were found to be associated with a poor response to vaccination in KTRs (23), and an increased CD4/CD8 ratio was determined to predict the vaccine response in patients with human immunodeficiency virus (27). Nevertheless, in our cohort, there was no significant correlation between the lymphocyte profiles (total CD3 and CD4 counts and CD4/CD8 ratio) and the immune response, including both humoral and cellular components (**Supplemental Figure 1**, **Supplemental Figure 2**).

### Fluctuation in renal function after vaccination

**Figure 3:**
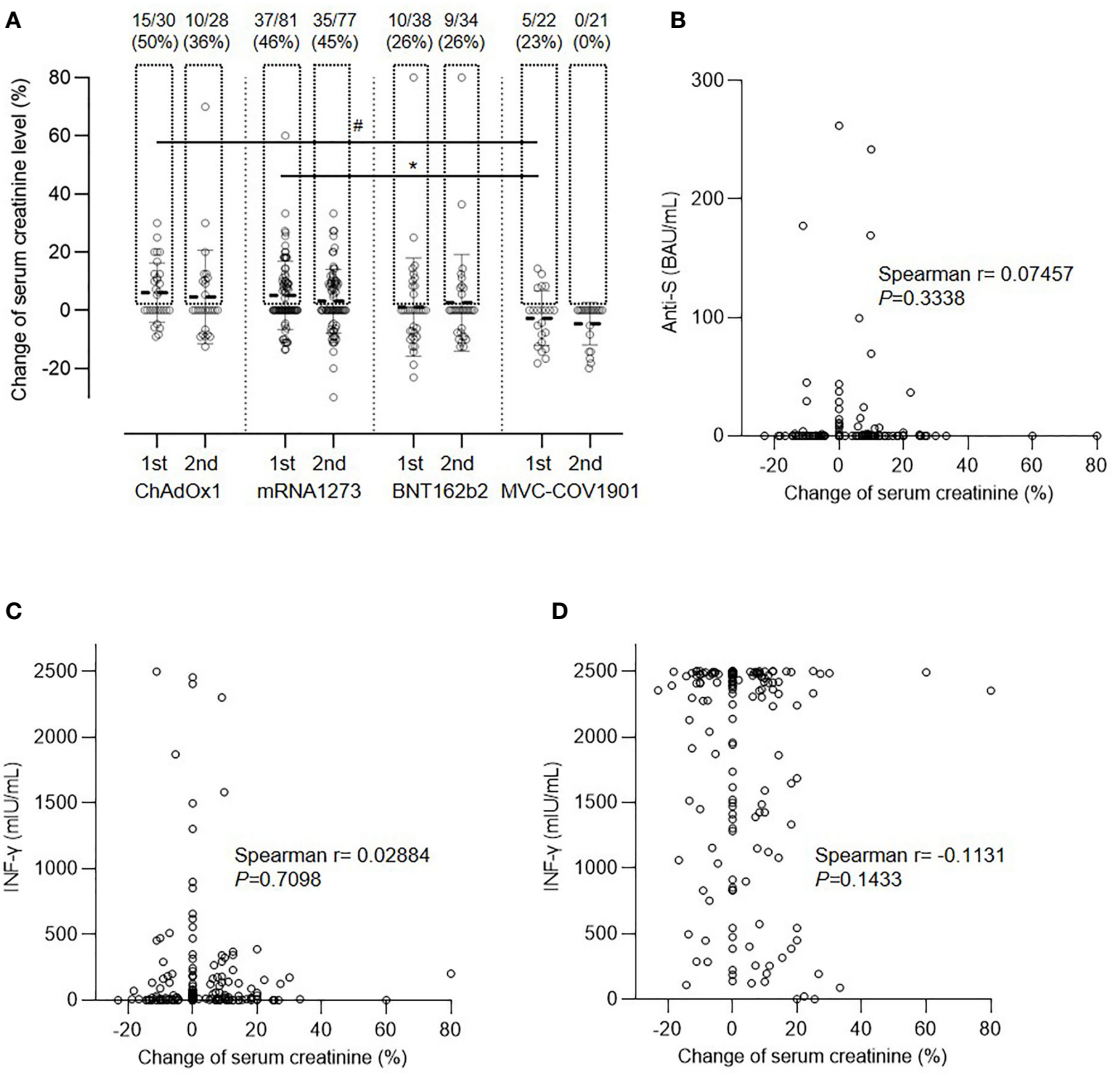
Change in serum creatinine level after vaccination. **(A)** Comparison of creatinine level after the first and second doses of the four vaccines, ^#^ *P*=0.0606, *P=0.0471. **(B)** Correlation between creatinine change and anti-spike protein antibody (anti-S) level. **(C)** Correlation between creatinine change and spike protein specific interferon-γ (IFN-γ) level. **(D)** Correlation between creatinine change and interferon-γ (IFN-γ) level after nonspecific stimulation.

After vaccination with the first dose, elevated creatinine levels were observed in some patients (**Figure 3A**). Approximately half of the vaccinated patients experienced a deterioration in renal function within 2 months after the first dose of ChAdOx1 (50%) and mRNA1273 (46%), whereas patients in the BNT162b2 and MVC-COV1901 groups presented with less fluctuation in serum creatinine levels (26% and 23%, respectively; P=0.0450 for the comparison among the four groups). The mean elevations in creatinine levels for ChAdOx1, mRNA1273, BNT162b2, and MVC-COV1901 were 6.06 ± 10.17, 5.09 ± 11.73, 0.72 ± 16.52, and −2.75 ± 9.35%, respectively (P=0.0213). The change in creatinine levels was attenuated after the second dose as follows: 4.57 ± 16.06, 3.08 ± 10.89, 2.58 ± 16.66, and −4.62 ± 7.30%, respectively (P=0.0721). Fluctuations in creatinine levels did not correlate with either humoral (**Figure 3B**) or cellular responses (**Figure 3C**) to the spike protein after the first vaccination. In addition, the capacity of the T cell response to mitogens was reduced in immunosuppressed patients in comparison to that in the general population. Moreover, the response to nonspecific stimulation by mitogens was impaired, and the response did not correlate significantly with creatinine level fluctuations (**Figure 3D**; P=0.1537).

### Indication biopsies after vaccination

**Figure 4:**
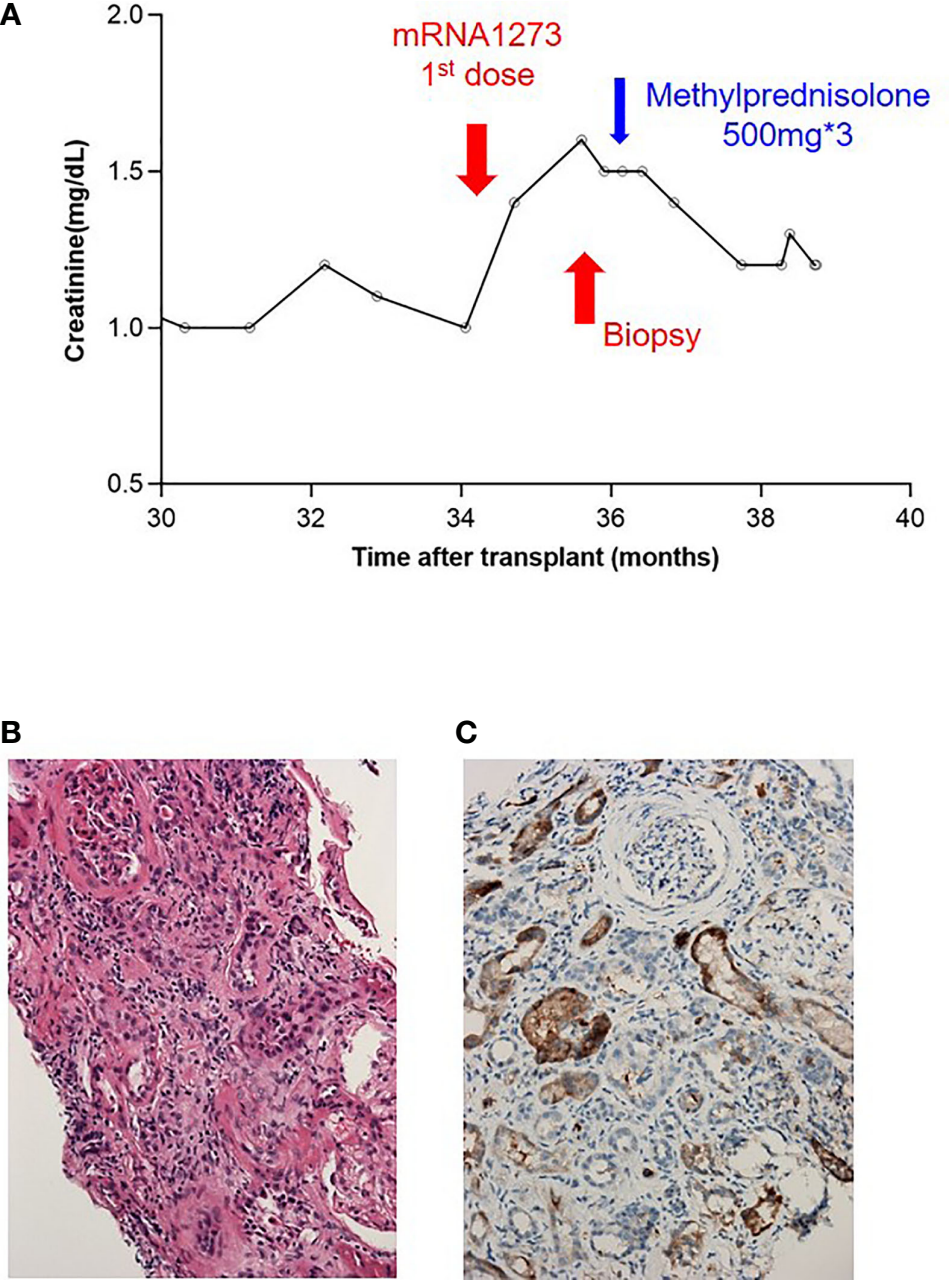
Presentation of a patient with borderline rejection after vaccination. **(A)** Serum creatinine level before and after vaccination. **(B)** H&E staining: mild tubulitis and some inflammatory cell infiltration in the interstitium with mild peritubular capillaritis. **(C)** C4d immunostaining: C4d deposition in 5–9% of peritubular capillaries.

In our cohort, the elevated serum creatinine level was mostly transient. We performed kidney biopsies on four patients (**Supplemental Table 4**) who sustained a creatinine elevation >20% for >2 months. Three of them had an etiology that could not be attributed to the effects of vaccination. Additionally, a 66-year-old female patient whose creatinine level reached 1.6 mg/dL from 1.0 mg/dL within 6 weeks (**Figure 4A**) after the first dose of mRNA1273 had mild interstitial inflammation, tubulitis, glomerulitis, peritubular capillaritis, and mildly increased C4d positivity in peritubular capillaries. The Banff scores of the renal biopsy were i1, t1, g1, ptc1, and C4d1 (**Figures 4B, C**); however, the patient underwent a 2-year protocol biopsy approximately year prior, and negative results were obtained for all categories of the Banff score. After steroid pulse therapy, her creatinine level was maintained at approximately 1.2 mg/dL.

## Discussion

In this observational study, KTRs received one of four different SARS-CoV-2 vaccines based on three platforms. Both cellular and humoral responses after vaccination were monitored. After repeated dosing, all platforms were immunogenic and evoked an immune response in some of the KTRs. mRNA1273 vaccination produced the best antibody detection rate and antibody titer. For the cellular response, mRNA1273 still resulted in the highest IFN-γ concentration among the patients with detectable responses. Different platforms were associated with distinct characteristics in KTRs. mRNA vaccines, especially mRNA1273, had higher immunogenicity than vector and subunit vaccines. Notably, cellular responses evoked by ChAdOx1 remained unchanged after the second dose, both in terms of the positive rate and IFN-γ concentration. In line with immunogenicity, some patients presented with deteriorating renal function after vaccination, particularly with mRNA1273 and ChAdOx1. In contrast, most KTRs receiving MVC-COV1901 had more stable renal function despite a reduced immune response. The fluctuation of creatinine levels, however, was self-limited and transient in most patients, and there was only one case of biopsy-proven borderline rejection after mRNA1273 vaccination without distinct causality. We speculated that this phenomenon might be caused by the “bystander” effect resulting from systemic inflammation. Nevertheless, the immune response to the spike protein did not correlate with the change in serum creatinine levels. Further investigation is needed to reach a definite conclusion regarding causality. Hence, different vaccines are associated with distinct safety and efficacy considerations for KTRs.

Viral vector-based vaccines use replication-deficient adenoviral vectors that are engineered to express the gene encoding the SARS-CoV-2 spike protein in infected cells, thus inducing an immune response to the SARS-CoV-2 spike protein (28). The mRNA vaccines were developed as alternatives to conventional vaccines because of their potential for rapid and flexible mass production (29). After the injection of nucleic acid-based vaccines, including viral vector-based and mRNA vaccines, the contents are delivered into the cytosol, resulting in expression of the spike protein in the transfected cells, inducing both cellular and humoral immune responses (30). The activity of transfected nucleic acids remains in the muscle for at least 2 months (31). Transfected mRNA and viral vectors are inherently immunostimulatory and can be recognized by a variety of cell surface, endosomal, and cytosolic immune receptors, resulting in the production of type I IFN, which might cause the rejection of allografts or relapse of glomerulonephritis (15, 17). In addition to inducing immune responses to the spike protein, the immune system also attacks the transfected cells expressing the spike protein, resulting in the destruction of transfected cells, including adipocytes, fibroblasts, and muscle cells (32, 33). The destruction of transfected muscle cells might be another reason for elevated serum creatinine levels in patients after vaccination with mRNA or viral vector-based vaccines without evidence of rejection episodes or nephritis.

The major concern with replication-deficient viral vector-based vaccines is that the virus can become replication-competent in cells concurrently infected with replication-deficient vaccine adenovirus and wild-type adenovirus. A new replication-competent adenovirus can be assembled after the homologous recombination of genetic elements, resulting in severe adenovirus infection in renal transplant recipients, including allograft nephritis (34, 35). This might be another possible cause of deteriorated renal function after vaccination with viral vector-based vaccines. Despite the possible concerns with replication-deficient viral vector-based vaccines, their use in patients taking immunosuppressants is not considered a contraindication (36).

Protein subunit vaccines have also been developed as a safer alternative to counteract SARS-CoV-2, but they often require adjuvants to elicit an immune response. One benefit of the protein subunit vaccines is that they do not result in the expression of spike proteins in muscle cells at the injection site. Therefore, they do not attract immune cells to attack muscle cells.

We found that mRNA1273 had higher immunogenicity than BNT162b2 in both KTRs and healthy individuals. One study reported that mRNA1273 induces significantly higher levels of anti-spike receptor-binding domain IgG (37) than BNT162b2. Another research team in Belgium also reported significant differences in the anti-spike protein IgG levels in 1,647 healthy adults who received mRNA1273 or BNT162b2 and attributed this result to the increased mRNA content of mRNA1273 (38). Because each dose of mRNA1273 delivers 100 µg of mRNA, whereas each dose of BNT162b2 contains 30 µg, it was speculated that a higher dosage of mRNA could translate into a stronger immune response. Mateus et al. (39) compared the immune response to two different mRNA doses (25 µg versus 100 µg) of mRNA1273 and found that those who received higher mRNA doses had significantly higher anti-spike protein antibody levels. Both vaccines also differ in the lipid nanoparticles that are used to transfect mRNA into cells after injection into the human body, and there might be some compositional differences that could also contribute to the differences in the humoral response (40); specifically, ALC-0315 and SM-102 are the cationic lipid components of lipid nanoparticles in BNT162b2 and mRNA1273, respectively (40).

Notably, KTRs exhibit a poor response to SARS-CoV-2 vaccination for all vaccines (6). Antimetabolite agents are important risk factors (22, 23, 41), and our study confirmed this observation based on both humoral and cellular responses. MMF reduces B cell numbers and blocks both primary and secondary humoral responses to vaccination (42). In our study, MMF diminished antibody titers and impaired cellular responses. In contrast, tacrolimus levels had less of an effect on immune responses after vaccination. The modification of drug combinations might be necessary for individuals who have a poor response. Additionally, booster doses can be administered (8, 10, 43); however, their safety and association with organ rejection might be a concern (43, 44). Our study showed that certain patients had elevated creatinine levels after vaccination and reported one borderline rejection case; this patient was successfully treated with steroids. Of note, protein subunit vaccines might have lower risks than other vaccines as a booster. Nevertheless, more booster doses might be necessary owing to the decreased immunogenicity of these vaccines, but the safety of these vaccines must be further investigated.

There were several limitations in this study. First, owing to the status of the pandemic and availability of vaccines, the number and characteristics of patients in each group were not equally distributed. KTRs in the mRNA1273 group were older and had longer post-transplant intervals because they were prioritized when mRNA1273 was available in our country. Old age is a risk factor for a poor response, but longer post-transplant intervals are related to a higher seropositivity rate (22). These two factors were not significant in another cohort (23). In this study, we tried to minimize the bias with propensity score matching, and the results still led to a similar conclusion. Second, this was a single-center study in an Asian country, and it is unclear if race has an effect on vaccination. Third, the assay for the immune response in our study was limited to spike protein-specific reactions. Moreover, the virus neutralization ability was not measured. Further, seroprotective thresholds for breakthrough infections could not be identified because of the low COVID-19 prevalence rate in our country. Further data collection for breakthrough infections and the waning of immune responses is needed.

In conclusion, we have shown the distinctive characteristics of four SARS-CoV-2 vaccines based on three platforms in KTRs. All vaccines had the capacity to evoke an immune response in the KTRs after repeated doses. mRNA vaccines, especially mRNA1273, are immunogenic. Clinicians should pay careful attention to renal function after vaccination, particularly when using the mRNA- and vector-based platforms. The traditional subunit vaccine induced a low immune response and had less of an effect on serum creatinine levels. These findings might help determine future COVID-19 vaccination strategies in KTRs and address other pathogenic targets.

## Data availability statement

The original contributions presented in the study are included in the article/**Supplementary Material**. Further inquiries can be directed to the corresponding authors.

## Ethics statement

The studies involving human participants were reviewed and approved by Research Ethics Committee of the National Taiwan University Hospital NTUH: 202106046RINA. The patients/participants provided their written informed consent to participate in this study.

## Author contributions

C-CC, M-KT, and C-YL conceived and designed the experiments. C-CC, Y-JH, M-JL, M-HL, W-CL, H-YL, Y-CL, Y-TH, and Y-FL performed the experiments. C-CC, Y-JH, M-JL, and C-YL analyzed the data. W-CL, Y-TH, and Y-FL contributed reagents. C-CC and C-YL drafted the paper. C-CC, W-CL, M-KT, and C-YL contributed to the discussion. All authors contributed to the article and approved the submitted version.

## Funding

C-CC and C-YL were supported by the National Taiwan University Hospital (MM022-2). C-CC was also supported by Academia Sinica (AS-KPQ-109-BioMed, AS-SUMMIT-109, and MOST-108-3114-Y-001-002) and E-Da Hospital (EDPJ 110065 and 110077).

## Conflict of interest

The authors declare that the research was conducted in the absence of any commercial or financial relationships that could be construed as a potential conflict of interest.

## Publisher’s note

All claims expressed in this article are solely those of the authors and do not necessarily represent those of their affiliated organizations, or those of the publisher, the editors and the reviewers. Any product that may be evaluated in this article, or claim that may be made by its manufacturer, is not guaranteed or endorsed by the publisher.
