# Supplementary material for: Immunogenicity and safety of two-dose SARS-CoV-2 vaccination via different platforms in kidney transplantation recipients
Source: Front Immunol. 2022 Sep 16;13:951576. doi: 10.3389/fimmu.2022.951576 (PMC9523367; doi:10.3389/fimmu.2022.951576)
Supplement: Supplementary file 1 [file DataSheet_1.docx]

Supplemental material

Supplemental Table 1. Comparison between group ChAdOx1 and mRNA1273 after propensity score matching

|  | ChAdOx1  (n=23) | mRNA1273  (n=23) | *P* value |
| --- | --- | --- | --- |
| Age (years) | 53.01±9.99 | 55.41±12.18 | 0.4680 |
| Male, n (%) | 10 (43.48) | 10 (43.48) | >0.9999 |
| Time since transplant (years) | 6.42±5.34 | 8.87±6.96 | 0.1879 |
| Creatinine (mg/dL) | 1.17±0.36 | 1.36±0.90 | 0.3391 |
|  |  |  |  |
| Anti-spike protein antibody |  |  |  |
| Positive rate after 1^st^ dose (%) | 4.35 | 22.73 | 0.0959 |
| Positive rate after 2^nd^ dose (%) | 37.93 | 62.07 | 0.0546 |
| Antibody titer for positive patients (BAU) | 277±351 | 2357±2322 | 0.0155 |
| Anti-spike protein IGRA |  |  |  |
| Positive rate after 1^st^ dose (%) | 36.36 | 30.00 | 0.7499 |
| Positive rate after 2^nd^ dose (%) | 38.10 | 42.86 | >0.9999 |
| IFN level for positive patients (mIU/mL) | 475.7±410.4 | 1443±919.9 | 0.0206 |

IGRA: interferon-γ release assay; IFN: interferon-.

Supplemental Table 2. Comparison between group BNT162b2 and mRNA1273 after propensity score matching

|  | BNT162b2  (n=27) | mRNA1273  (n=27) | *P* value |
| --- | --- | --- | --- |
| Age (years) | 52.68±11.46 | 54.69±10.94 | 0.5131 |
| Male, n (%) | 11 (40.74) | 7 (25.93) | 0.3869 |
| Time since transplant (years) | 8.76±7.51 | 8.59±7.32 | 0.9362 |
| Creatinine (mg/dL) | 1.10±0.28 | 1.17±0.82 | 0.6863 |
|  |  |  |  |
| Anti-spike protein antibody |  |  |  |
| Positive rate after 1^st^ dose (%) | 14.81 | 23.08 | 0.5007 |
| Positive rate after 2^nd^ dose (%) | 52.00 | 76.00 | 0.1398 |
| Antibody titer for positive patients (BAU) | 465.6±687.2 | 2907±2288 | 0.0047 |
| Anti-spike protein IGRA |  |  |  |
| Positive rate after 1^st^ dose (%) | 22.22 | 28.00 | 0.7523 |
| Positive rate after 2^nd^ dose (%) | 43.48 | 41.67 | >0.9999 |
| IFN level for positive patients (mIU/mL) | 597.0±691.7 | 1673±900.6 | 0.0115 |

IGRA: interferon-γ release assay; IFN: interferon-.

Supplemental Table 3. Comparison between group MVC-COV1901 and mRNA1273 after propensity score matching

|  | MVC-COV1901  (n=16) | mRNA1273  (n=16) | *P* value |
| --- | --- | --- | --- |
| Age (years) | 51.08±10.12 | 51.96±11.09 | 0.8163 |
| Male, n (%) | 5 (31.25) | 9 (56.25) | 0.2852 |
| Time since transplant (years) | 10.85±7.19 | 12.97±9.02 | 0.4670 |
| Creatinine (mg/dL) | 1.31±0.46 | 1.45±1.08 | 0.6507 |
|  |  |  |  |
| Anti-spike protein antibody |  |  |  |
| Positive rate after 1^st^ dose (%) | 6.25 | 40.00 | 0.0373 |
| Positive rate after 2^nd^ dose (%) | 33.33 | 80.00 | 0.0253 |
| Antibody titer for positive patients (BAU) | 509.5±1112 | 2302±1891 | 0.0186 |
| Anti-spike protein IGRA |  |  |  |
| Positive rate after 1^st^ dose (%) | 18.75 | 28.57 | 0.6746 |
| Positive rate after 2^nd^ dose (%) | 42.86 | 35.71 | >0.9999 |
| IFN level for positive patients (mIU/mL) | 344.5±274.5 | 1200±1166 | 0.1775 |

IGRA: interferon-γ release assay; IFN: interferon-.

Supplemental Table 4. Patients undergoing to indication biopsy after SARS-CoV-2 vaccination

|  | 1 | 2 | 3 | 4 |
| --- | --- | --- | --- | --- |
| Age (year) | 69 | 42 | 57 | 53 |
| Sex | F | M | M | F |
| Vaccine | mRNA1273 | BNT162b2 | ChAdOx1 | ChAdOx1 |
| Creatinine (mg/dL) |  |  |  |  |
| Before vaccination | 1.0 | 1.6 | 1.7 | 1.0 |
| After vaccination | 1.6 | 2.4 | 3.7 | 1.9 |
| Banff score |  |  |  |  |
| ti | 1 | 0 | 0 | 0 |
| i | 1 | 0 | 0 | 0 |
| i-IFTA | 1 | 0 | 0 | 0 |
| t | 1 | 0 | 0 | 0 |
| v | 0 | 0 | 0 | 0 |
| g | 1 | 0 | 0 | 0 |
| ptc | 1 | 0 | 0 | 0 |
| C4d | 1 | 1 | 1 | 2 |
| Diagnosis | Borderline TCMR  Suspected ABMR | Diabetic nephropathy | Negative | Arteriosclerosis |
| Final creatinine | 1.2 | 2.3 | 1.9 | 2.2 |
| (Time after biopsy, months) | 2 | 1 | 1 | 1 |

TCMR: T-cell mediated rejection; ABMR: antibody-mediated rejection.

Supplemental Figure 1. Correlation between antibody (anti-S) levels and T lymphocytes: (A) total T cell count; (B) CD4+ T cell counts; (C) CD8+ T cell counts; (D) ratio of CD4+ T cell count/CD8+ T cell count

Supplemental Figure 2. Correlation between IFN-γ level and lymphocytes: (A) total T cell count; (B) CD4+ T cell counts; (C) CD8+ T cell counts; (D) ratio of CD4+ T cell count/CD8+ T cell count
