# Supplementary material for: Immunogenicity and safety of two-dose SARS-CoV-2 vaccination via different platforms in kidney transplantation recipients
Source: Front Immunol. 2022 Sep 16;13:951576. doi: 10.3389/fimmu.2022.951576 (PMC9523367; doi:10.3389/fimmu.2022.951576)

Supplemental Figure 1. Correlation between antibody (anti-S) levels and T lymphocytes: (A) total T cell count; (B) CD4+ T cell counts; (C) CD8+ T cell counts; (D) ratio of CD4+ T cell count/CD8+ T cell count

Supplemental Figure 1A

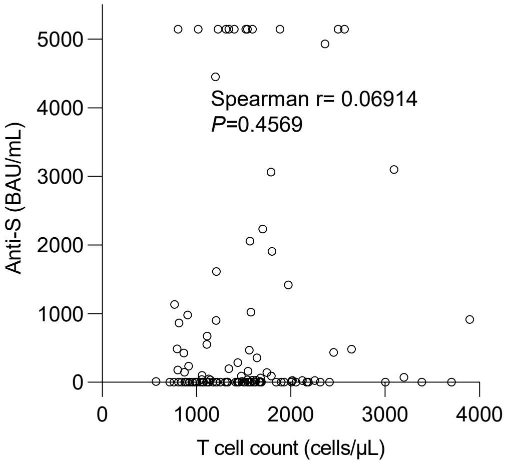

Supplemental Figure 1B

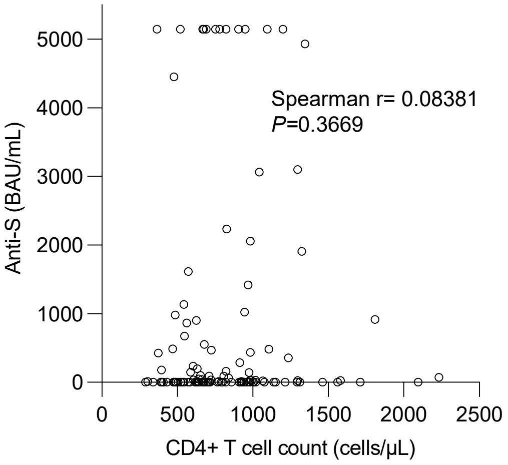

Supplemental Figure 1C

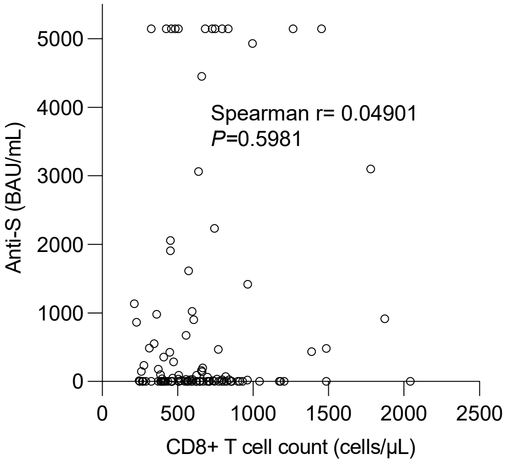

Supplemental Figure 1D

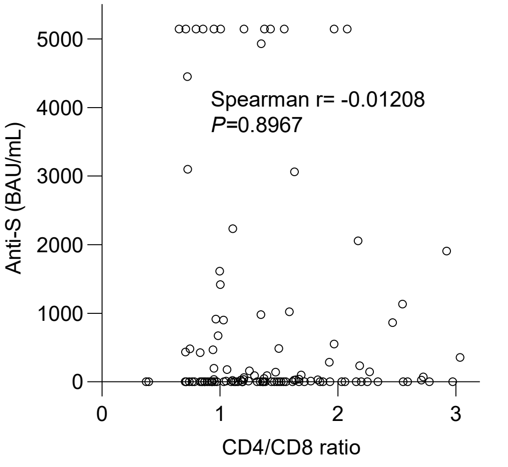

Supplemental Figure 2. Correlation between IFN-γ level and lymphocytes: (A) total T cell count; (B) CD4+ T cell counts; (C) CD8+ T cell counts; (D) ratio of CD4+ T cell count/CD8+ T cell count

Supplemental Figure 2A

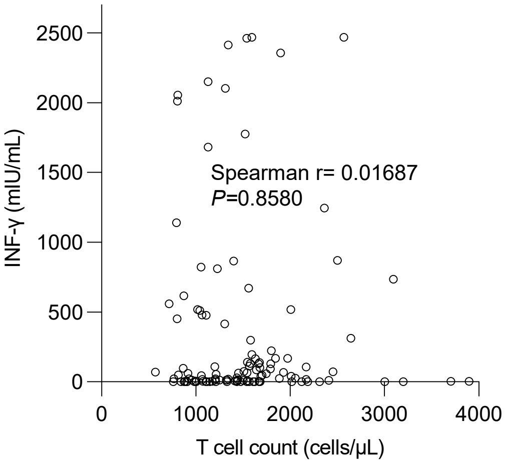

Supplemental Figure 2B

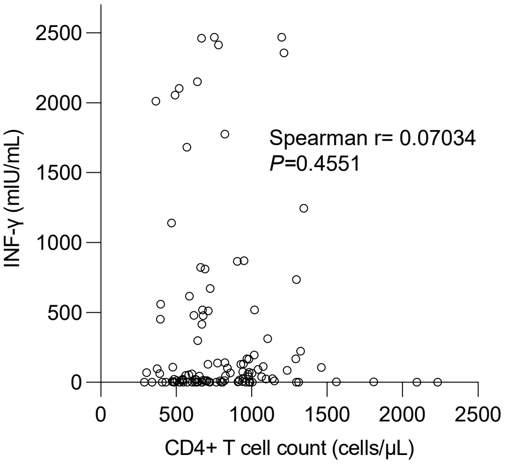

Supplemental Figure 2C

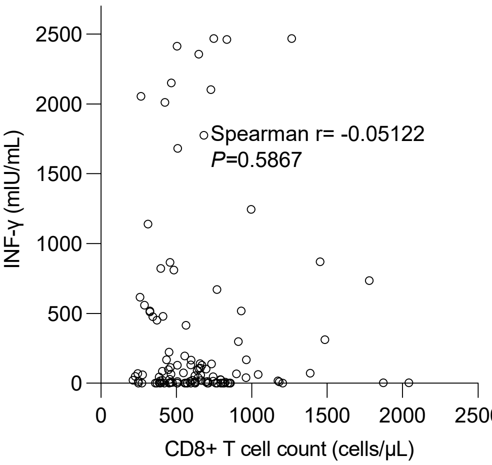

Supplemental Figure 2D

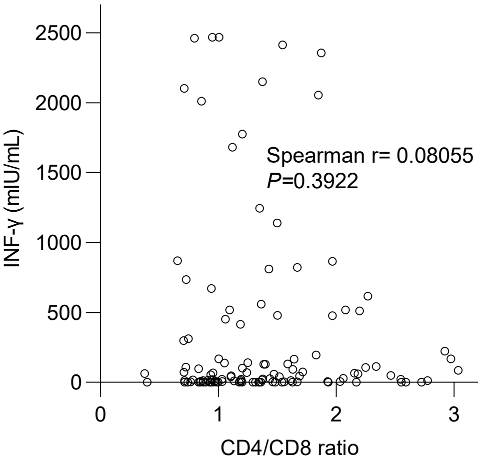

Supplement: Supplementary file 2 [file Image_1.pdf]
